# Retention of Biomedical Knowledge in Medical Students Predicts USMLE Step 1 Scores

**DOI:** 10.15694/mep.2021.000070.1

**Published:** 2021-03-12

**Authors:** Anna Blenda, Renee Chosed, Carrie Bailes, Mary Caldwell, Matthew Tucker

**Affiliations:** 1 University of South Carolina School of Medicine Greenville

**Keywords:** Memory retention, Biochemistry, USMLE Step 1, Free recall, Cued recall

## Abstract

This article was migrated. The article was marked as recommended.

Introduction

Medical students are tasked with learning a vast amount of medical knowledge prior to sitting for the USMLE Step 1 exam, a portion of which is either forgotten or becomes inaccessible to memory following each exam. In this study we examined whether accessibility and retention of 1 ^st^-year biochemistry content predicts performance on high stakes exams such as USMLE Step1.

Methods

First-year medical students were retested on a subset of biochemistry final exam items 10.5 months after sitting for the original exam. Retention was measured as a percentage of the original final exam score. Availability of information was measured with cued recall (i.e., selecting from a list the multiple-choice distractors), while accessibility of information was captured through free recall (without the aid of multiple choice distractors).

Results

As expected, we found that free recall rates were much lower than cued recall rates, but that students who scored higher on Step 1 had a smaller gap between cued and free recall scores, demonstrating a greater ability to access information than lower-scoring students. Importantly, we also demonstrate that higher-scoring students retained a higher percentage of the original biochemistry material than lower-performing students after 10.5 months, and that the amount information retained in memory was associated with higher scores on Step 1, demonstrating the potential importance of teaching medical school content with the intention of *making it stick*, especially in students who are not as strong academically.

Conclusion

The methods employed in this study are straightforward and can be used to compare retention and accessibility of information across medical school courses, and may serve as a guide to curriculum and pedagogical improvements.

## Introduction

Medical students absorb a vast amount of knowledge during their first two years of medical school prior to taking the USMLE Step 1 exam. Therefore, questions regarding how much information is retained with the passage of time, and how accessible that information is, should be of paramount importance in evaluating the effectiveness of the curriculum and pedagogical techniques in preparing students for important benchmarks such as USMLE Step 1 and for careers as physicians.

During the pre-clinical years of medical school, a common strategy which students employ to deal with large amounts of information is to cram for final exams so that the information is accessible at the time of the test. Under this circumstance, however, it is likely that substantial amounts of information will fall into disuse and be forgotten as a new module/course starts and new material must be learned. This leaves faculty with a set of module grades that reflects students’ knowledge in the moment, with little understanding about whether that information will be retained over the long term. One way to better understand how well the material was initially learned is to utilize direct measures of retention. For example, if medical students retake a multiple-choice question (MCQ) final exam after a one-year delay and the average score drops from 82% to 46%, those students have retained 56% of the material that was originally learned and that was deemed important for students to know ahead of the Step 1 exam and clinical clerkships.

Retention rate can be used as a benchmark to devise pedagogic strategies and course content improvements to increase retention in modules and to identify areas of relative difficulty within and across modules. The implication of using such a measure should not be underestimated, as the retention rate can help faculty understand whether too much is being taught, or whether the content is too complex or inadequately covered. While several studies have looked at biomedical sciences knowledge cross-sectionally and over different time spans, there have been comparatively few that have used direct measures of retention (test-retest) of the same material tested at different times ( Custers, 2010), and no studies to our knowledge have examined the association between retention rates and performance on high-stakes standardized medical school exams (i.e., USMLE Step 1).

Research on retention of biomedical sciences knowledge shows that knowledge retention over time depends on the nature of the material. Published studies showed that one-year retention rates on MCQ-style exams vary based on the nature of the content: physiology: 80%, neuroanatomy: 47.5%, and immunology: 80% ( D’Eon, 2006), anatomy: 75% ( Blunt and Blizard, 1975), and pharmacology: >100% after 8 months ( Rodriguez *et al.*, 2002) and after 22 months ( Saffran, Kennedy and Kelley, 1982). Relevant to the current study, the few studies that have assessed biochemistry knowledge using an MCQ-format, reveal retention rates of 70% after one year ( Rico, Galindo and Marset, 1981), 66% after 22 months ( Saffran, Kennedy and Kelley, 1981), and 66% after two years ( Kennedy, Kelley and Saffran, 1981). Another study found that students retained 78% of biochemistry content in the time between USMLE Step 1 and Step 2CK ( Ling *et al*. 2008). From these studies it is clear that content from different medical domains is differentially retained, highlighting that the use of such results could help guide faculty in their efforts to improve teaching methods and make adjustments to important factors such as cognitive load and the amount and spacing of content delivery ( Khalil and Elkhider, 2016).

Direct measures of biomedical knowledge retention over time are indeed important, and there are a handful international studies published in the last 50 years that have attempted in various ways document retention over time. One drawback of many studies is that assessment of memory relies on the use of MCQs ( Custers, 2010), where the answer to each question is embedded in a list of distractors (i.e., cued recall): the student need only recognize the correct answer from the list, as opposed to recalling the answer without cues. However, recalling information without the aid of cues (i.e., free recall) is an important measure of memory, one that physicians often rely on, which requires that the information is not only available (through cuing), but also accessible without cues.

One way to address this shortcoming of MCQ tests is to have students recall answers to test questions from memory before seeing the multiple choice options for the question. Free recall rates can be viewed as a measure of how well-instantiated the material was learned: not only is the information available in memory, but the student is also able to access the answer without the help of cues. Thus, cued recall (using MCQs) assesses whether the information was merely *available* for recall, while free recall requires the information to be available, but also *accessible* in the absence of cues ( Tulving and Pearlstone, 1966). Given the desirability of committing to memory information that is accessible (not merely available), it may be helpful for medical schools to help students improve their free recall of information, as it may be a better indicator that the information is ‘stuck’ in memory ( Brown, 2014).

While several studies have examined retention rates for a range of content areas, very little research has focused on different types of retention (free v. cued recall) or the relationship between retention and standardized measures of performance. In the present study participants were re-examined on a portion of one of the first of their first-year final exams (Molecular & Cellular Foundations of Medicine) after a 10.5 month delay to determine the retention rate for that information. The first part of each question required students to access the answer from memory without the aid of seeing the multiple choice distractors (free recall), while the second part of each question was meant to replicate past studies that had students select an answer from a list of MCQ distractors (cued recall). These measures of retention were then regressed against academic performance and USMLE Step 1 scores to assess their relationships to these important measures of student success during medical school. We hypothesized that cued recall scores on the original end-of-module exam and the research exam would be associated with higher overall class rank and Step 1 scores. We also expected free recall rates would be lower than cued recall rates, but that we would see a similar correlation between free recall of information on the research exam and these standardized measures. Importantly, we also hypothesized that knowledge retention over 10.5 months would also be associated with these measures.

## Methods

### Participants

Participants were 44 medical students (43% of the class) from the University of South Carolina School of Medicine Greenville (24 Females, Age: 24.7±1.7yrs, Range: 23-31yrs). Participants completed the research exam the day after completing the first module of their second year, a course on infectious diseases. Demographic data (age and sex), MCAT scores, Step 1 scores, and class rank were collected. This study was approved by the University of South Carolina Institutional Review Board.

### Research exam

Items from the final exam of the Molecular & Cellular Foundations of Medicine (Biochemistry) module were used to create the research exam for this study. The Foundations module is the first biomedical sciences module completed by the first-year medical students. Of the 104 questions on the original exam, 50 questions were selected for the research exam that met the following criteria:

• Questions for which an answer could be recalled from memory without seeing the answer choices.
• Questions that were not overly difficult (>60% of students got the question correct).
• Questions that were good discriminators of student performance based on a point-biserial correlation: r>0.15.

**Figure 1.**
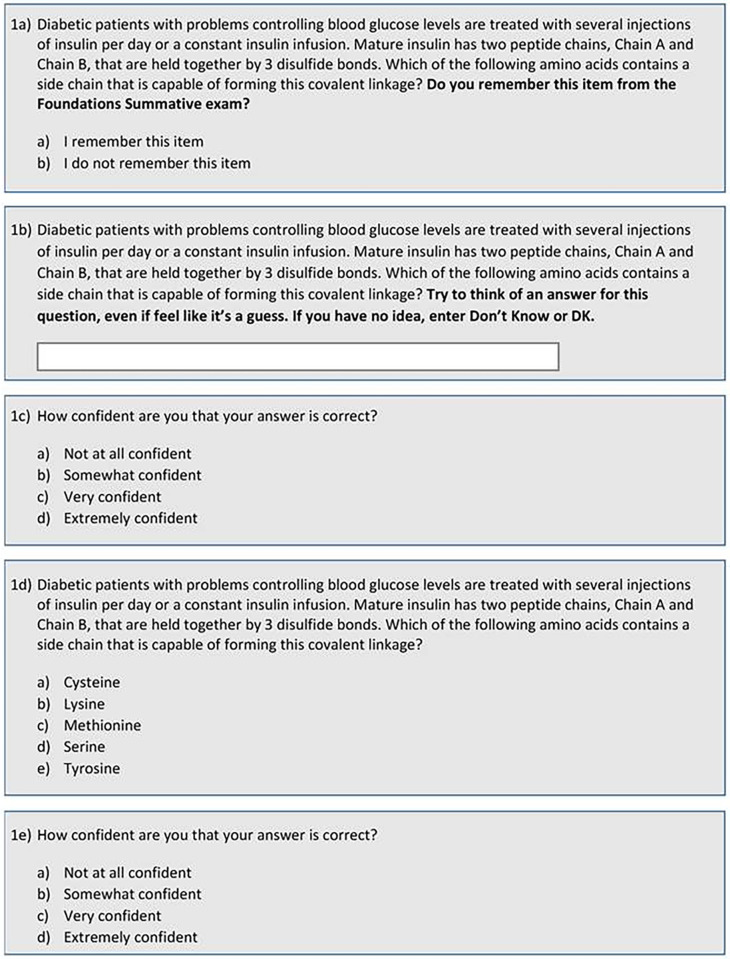
Example item. Each test item was divided into five parts to assess cued and free recall as well as confidence ratings.

Each of the 50 test items was presented in five parts (see Figure 1):

• Questions stem followed by the question: “Do you remember this item from the Foundations Summative exam?”
• Questions stem followed by: “Try to think of an answer for this question, even if you feel like it’s a guess? If you have no idea, enter Don’t Know or DK”. Participants entered their answers in a text box below the question stem.
• “How confident are you that your answer is correct?” (1-4 Likert scale)
• Question stem followed by the answer choices that appeared on the original exam.
• “How confident are you that your answer is correct?” (1-4 Likert scale)

The only change made to a question stem was to modify the last sentence to make it answerable for the free recall portion of each question:

*Free recall:* Based on the data presented, *what enzyme* is likely deficient in this patient?

*Cued Recall:* Based on the data presented, *which of the following enzymes* is likely deficient in this patient? (italics added)

The difficulty of each item was rated on a 1-10 scale (1=Very easy, 10=Very difficult) by content experts: the directors (lead faculty) of the Biochemistry Foundations module (RJC and AVB).

### Procedure

Participants completed the research exam in the same lecture hall used for the original Biochemistry summative exam and used the same software platform (ExamSoft: ExamSoft Worldwide, Inc). They were unaware of the nature and content of the test prior to participating. Upon arrival at the testing location at 10am, participants logged in to Examplify software on PC laptop computers and opened the exam. Prior to taking the test, participants completed a five-question Biochemistry Questionnaire in Examplify:

1. How knowledgeable were you about biochemistry prior to entering medical school?

a) Almost no knowledge of biochemistry

b) Some knowledge of biochemistry

c) A fair amount of knowledge about biochemistry

d) A strong knowledge of biochemistry

2. What percentage of Foundations content do you think you still remember (0-100%)? Be honest.

3. What score do you think you would get if you re-took your Foundations Summative exam (0-100)?

4. On a scale from 1-10 (1 = hate biochem, 10 = love biochem), how much do you enjoy biochem?

The exam started after completion of these questions. As with their original end-of-module exam, students were allowed to use the restroom, and two people (research assistants) were available to answer questions. The exam took approximately one hour to complete.

### Statistical Analyses

The primary variables of interest were percent correct on the original summative and research exam, memory retention (% retention: research exam score - original summative score/original summative score*100), and students’ USMLE Step 1 scores. Pearson correlations and partial correlations were conducted. Statistical significance was set to p<0.05.

## Results/Analysis

### Student Data

Preliminary analyses were performed to show that participants in this study were representative of the class as a whole. Students who participated in this study performed the same as their non-participant counterparts in the same class on the original Biochemistry summative exam (participants: 86.2±1.0%, non-participants (n=59): 86.4±0.9%, t _101_=0.15, p=0.88). Participants and non-participants were also similar in class rank at the end of the biochemistry module (participants: 54.1±4.8 (in a class of 103 students), non-participants: 51.9±3.9, t _101_=0.36, p=0.72). Participants did not perform better on the research exam if they reported remembering items from the original Biochemistry summative exam (remembered: 37.1±2.6 vs. not remembered: 34.7±2.3 items correct, paired-samples t-test, t _44_=0.91, p=0.37). As expected, class rank was strongly associated with all measures of performance, including USMLE Step 1 (r=-0.75, p<0.0001). To a lesser extent MCAT scores also predicted Step 1 performance (r=0.39, p=0.01).

### Exam performance at Time 1 and Time 2: Cued (MCQs) and Free Recall (No Cues)

**Figure 2.**
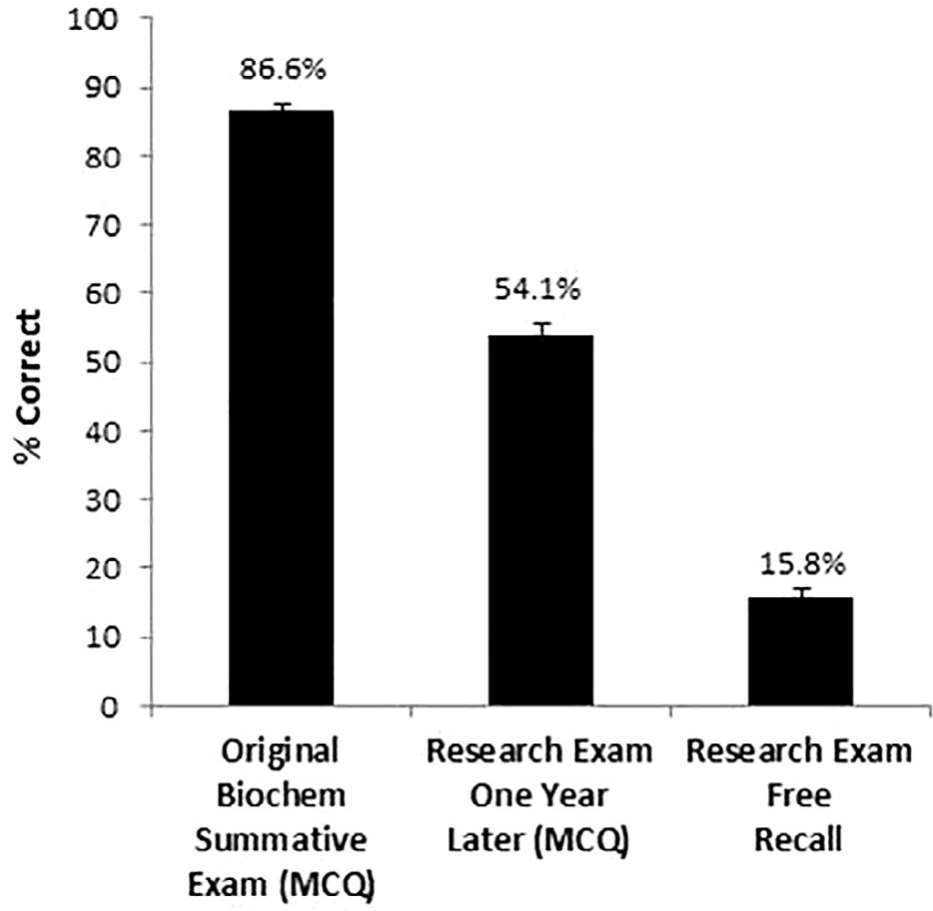
Test performance. Original summative score based on multiple choice questions (cued recall); Research exam score on same items from the original exam (cued recall); Research exam free recall percent correct.

*Cued Recall:* On the original exam, students scored 86.2±6.8% (SD) on the 50 items used in the research exam, and scored 54.6±1.4% on the research exam on these same items 10.5 months later. Thus, students retained 62.2±8.6% (SD) of the original biochemistry information over the 10.5-month interval ( Figure 2). As expected, expert difficulty ratings for each item correlated with the number of students getting an item correct, with fewer students getting the more difficult items correct (original exam: r=-0.29, p=0.05, research exam: r=-0.39, p=0.006). Participants also reported higher confidence ratings for cued recall answers that were correct on the original exam (r=0.46, p=0.002) and the research exam (r=0.64, p<0.001).

**Figure 3.**
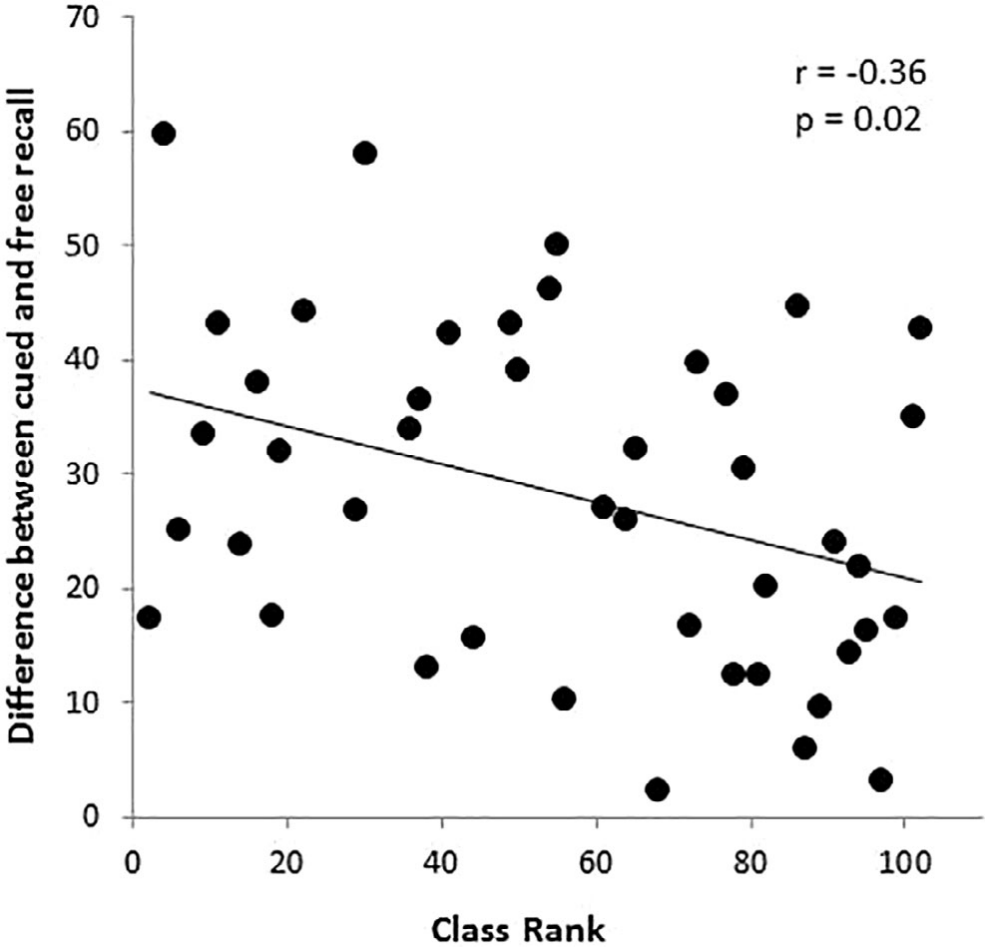
The relationship between USMLE Step 1 scores and the gap between cued and free recall.

With regard to academic standing, we found a marginal association between class rank at the time of the research exam and cued recall retention (r=-0.28, p=0.07), suggesting that academically stronger students retain more information. A relationship was also observed between class rank and the difference between cued recall and free recall on the research exam (% difference: r=-0.36, p=0.02), demonstrating that the gap between cued and free recall is smaller for stronger students ( Figure 3).

*Free recall:* Students recalled only 15.8±9.3% (SD) of the items correctly without aid of MCQ distractors ( Figure 2). However, we also found that free recall correlated with Step 1 scores (r=0.45, p=0.003), similar to the correlation for cued recall, which is presented below. We found that class rank was associated with higher confidence ratings for free recall answers (r=0.46, p=0.002), but not higher confidence in cued recall answers (r=-0.18, p=0.23).

### The relationship between memory retention and USMLE Step 1 performance

**Figure 4.**
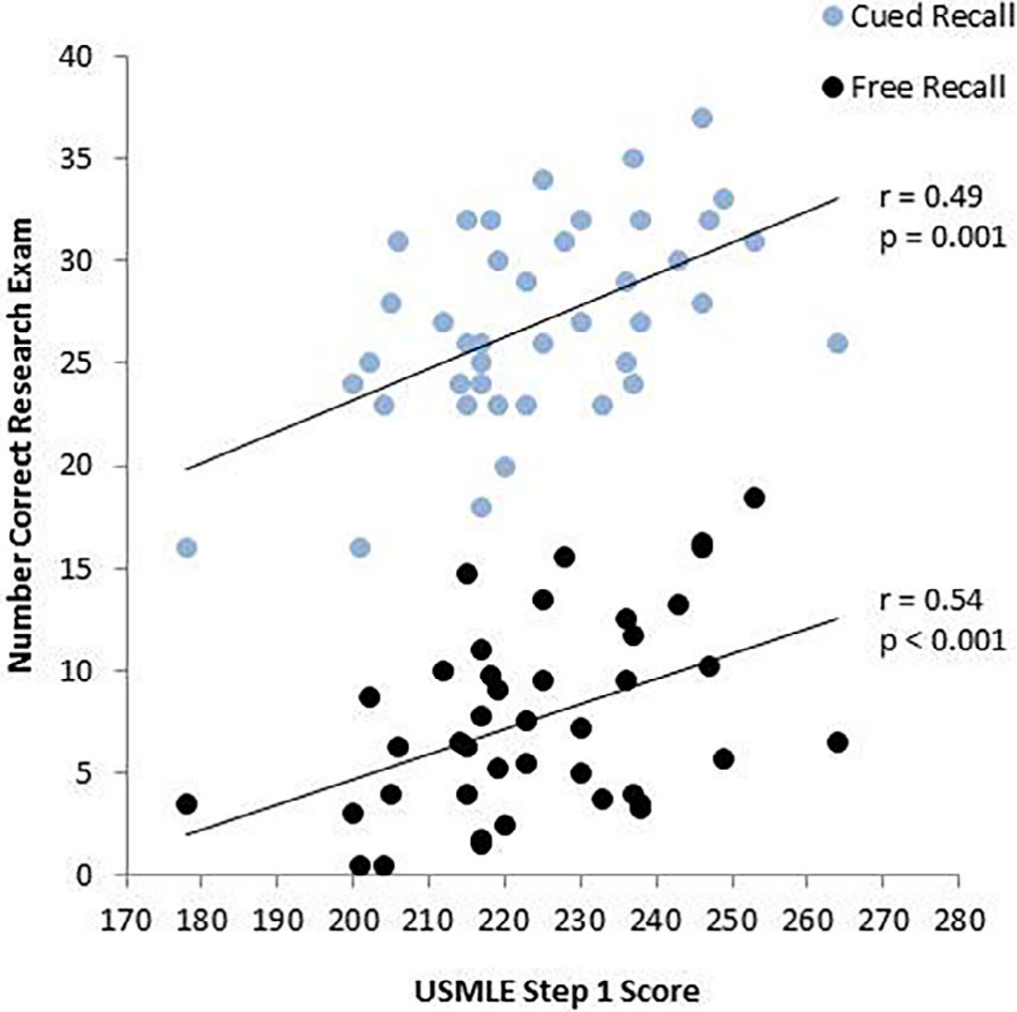
The relationship between performance on the original summative and the research exam 10.5 months later, and USMLE Step 1 performance.

*Cued recall:* As expected, percent correct on the original summative exam and the research exam was associated with higher Step 1 test scores (summative exam: r=0.49, p=0.001, research exam: r=0.54, p<0.001; Figure 4).

**Figure 5.**
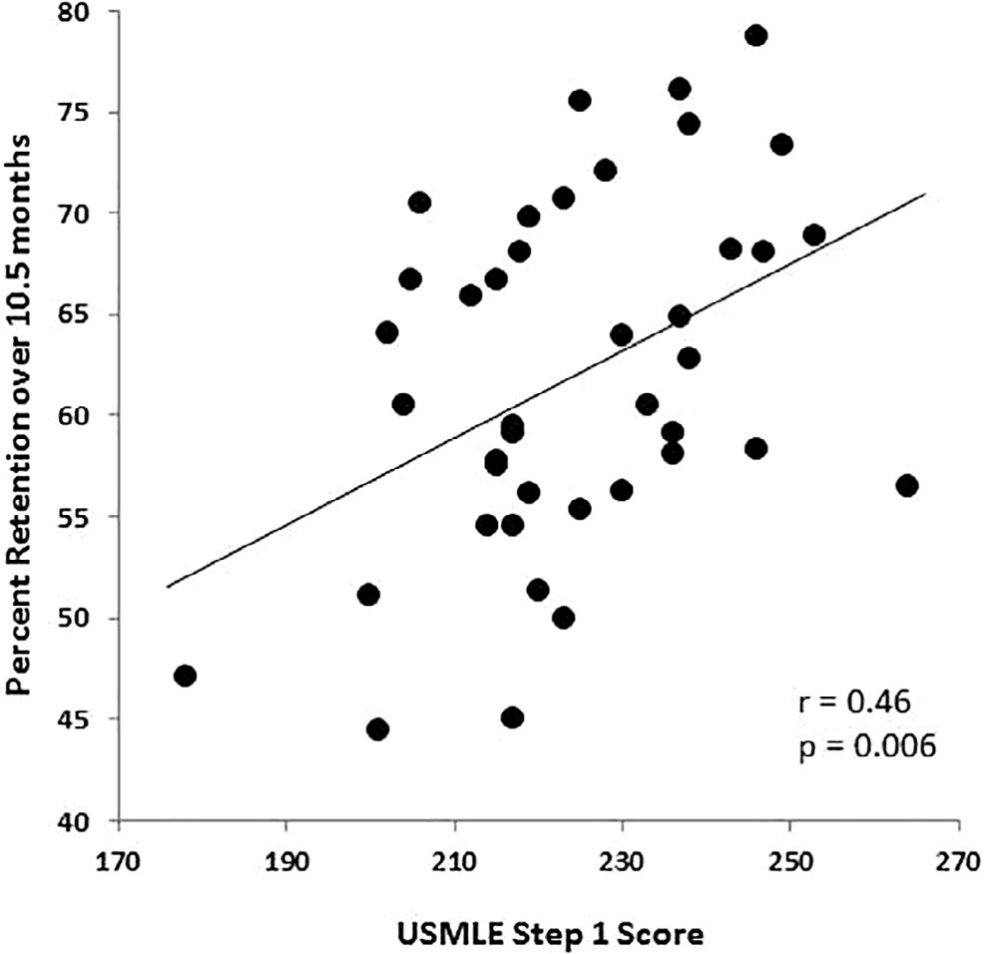
The relationship between retention (percent change in performance after 10.5 months) and USMLE Step 1 performance.

Importantly, however, the change in performance over 10.5 months (our measure of retention) also predicted standardized test scores: the amount of information retained in memory from the original summative exam to the research study after a 10.5-month delay was associated with higher USMLE Step 1 scores (r=0.42, p=0.006; Figure 5).

This correlation remained significant after conducting partial correlations to account for the influence of MCAT scores (Biological/ Biochemical Foundations of Living Systems subscore: r _partial_=.34, p=0.04), and MCAT total score: r _partial_=.36, p=.03), suggesting that this relationship is determined to a large extent by the knowledge gained during the first year of medical school, not the biochemistry knowledge with which students entered medical school. These associations, based on our measure of retention, were mirrored in students’ subjective estimates of how much they remembered from their Biochemistry Foundations module: participants who reported remembering more information performed better on Step 1 (r=0.30, p=0.055).

**Figure 6.**
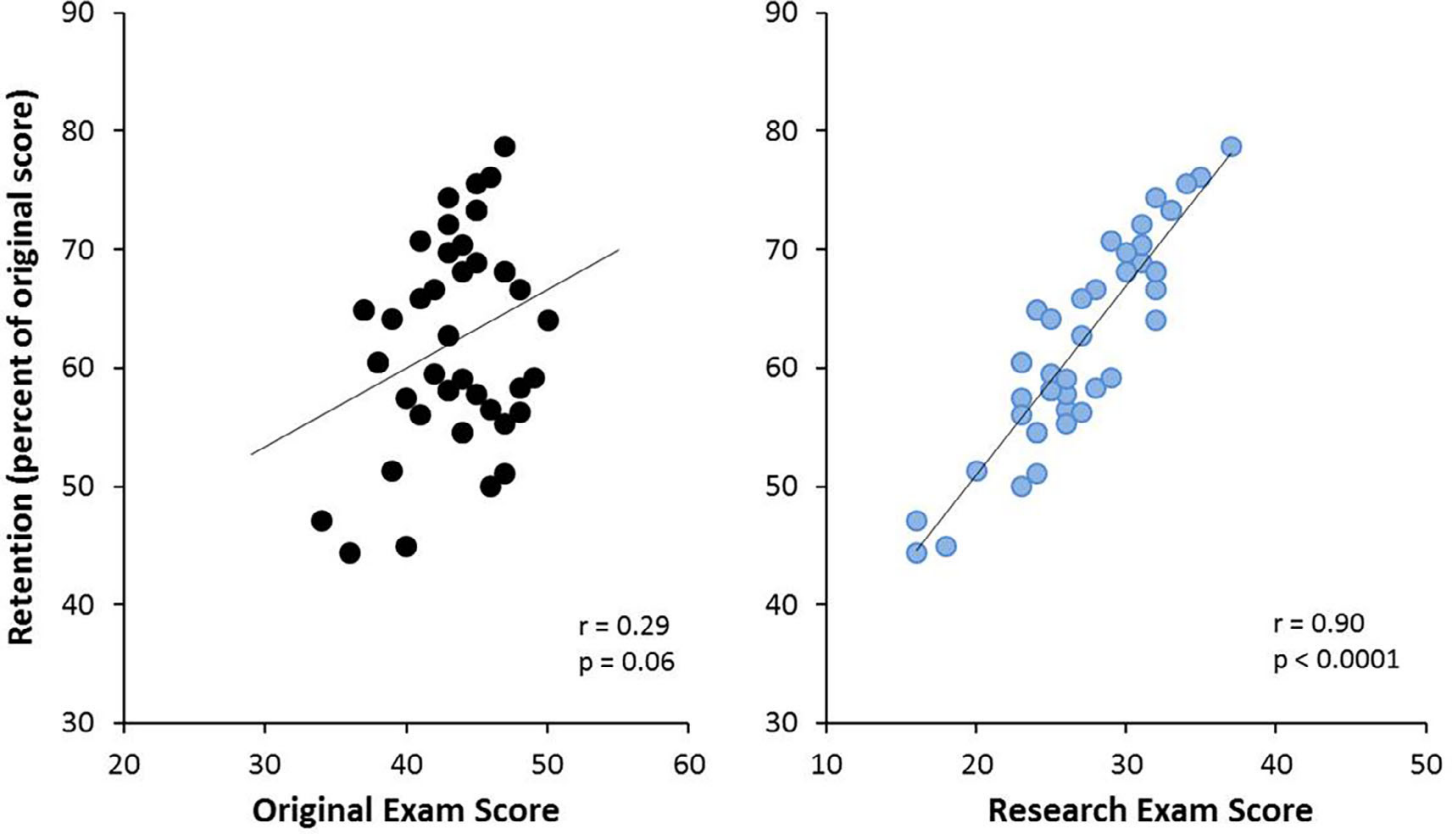
The relationship between retention, original summative exam score and research exam score.

Interestingly, the scores students obtained on the research exam were much more closely aligned with retention than scores on the original exam (r=0.90, p<0.0001, r=0.29, p=0.06, respectively; Figure 6). A Fisher’s Z transform and calculation for the difference between two dependent correlations resulted in a significant difference between these two correlations (Z=9.14, p<0.0001). This finding may suggest that the continued acquisition of knowledge from subsequent modules and repetition of existing knowledge over the 10.5-month delay may help better instantiate, and increase their knowledge of, the material originally learned during the Biochemistry module, for most all students.

### Subjective Data: Biochemistry Questionnaire Results

*Q1: Knowledge of biochemistry prior to entering medical school* - By subjective report, the average score for prior biochemistry knowledge was 2.2±0.6 (a little more than “Some knowledge of biochemistry”). Prior biochemistry knowledge did not correlate with performance on either exam, memory retention, MCAT subscales or total score, or Step 1 exam scores (all p values > 0.07).

*Q2: Percent remembered from the Foundations module* - Students estimated that they still remembered 35.3±16.5% of the content from the Biochemistry module. The association between students’ estimates of the amount they remembered and Step 1 scores were near-significant (r=0.30, p=0.06).

*Q3: What score do you think you would get if you re-took your Foundations Summative exam* - Participants estimated they would obtain a score of 49.0±14.6% (SD). Their actual score on the research exam was 54.6±9.7%, revealing that students had a tendency to underestimate the extent of their knowledge in biochemistry (paired-samples t-test, t _42_=2.22, p=0.03).

*Q4: How much do you enjoy biochemistry* - Participants rated their enjoyment of biochemistry (1 = “hate biochem”, 10 = “love biochem”) at 5.0±2.1. Higher enjoyment ratings were associated with higher MCQ scores (cued recall) on the original and research exam (r=0.33, p=0.04 and r=0.36, p=0.02, respectively), but did not quite reach significance for retention (r=0.27, p=0.08) or Step 1 scores (r=0.28, p=0.09).

## Discussion

In this study we found that after 10.5 months students retained 62.2% of their first-year biochemistry content as assessed with MCQs, which is in line with previous studies using a similar methodology ( Rico, Galindo and Marset, 1981; Saffran, Kennedy and Kelley, 1981; Kennedy, Kelley and Saffran, 1981). Not surprisingly, we also found that retention of information over 10.5 months was associated with scores on both exams, demonstrating that students who obtain higher scores also retain more information. This advantage in retention for stronger students also translated to higher Step 1 scores.

As expected, we found that cued recall performance on MCQs was much higher than free recall performance (55% vs. 15% on the research exam). While this result represents only one data point, it clearly indicates that free recall, a measure of memory performance requiring that information is both accessible and available to memory, is much more challenging for students. While the practical implications of both the free recall measure (15%) and the gap between free recall and cued recall score are difficult to interpret in the absence of comparative data, this finding provides a starting point for further research. It is likely that students who demonstrate a smaller gap between cued and free recall have better consolidated this information in memory. Finally, both the amount of information retained over the 10.5-month interval, as well as the cued and free recall scores, predicted performance on Step 1. Whether these correlations are weaker or stronger when examining other modules, or organ systems, and disciplines is still unknown. However, knowledge of these relationships could guide faculty in their efforts to improve the curriculum and pedagogic approaches to pre-clinical medical education.

Students’ subjective reports regarding biochemistry revealed a number of interesting associations. Those who reported greater enjoyment of biochemistry performed better on both exams and were somewhat more likely to retain more information over time and score higher on Step 1. Although the last two of these correlations did not quite reach significance, taken together they suggest that enjoyment, a measure of intrinsic motivation ( Ryan and Deci, 2000), is a student attribute that has an appreciable impact on performance. Conversely, self-reported biochemistry knowledge prior to medical school did not strongly predict MCAT or Step 1 scores and only marginally predicted scores on the original and research exam (0.05<p<0.10). This lack of association may simply be a result of students’ limited knowledge of biochemistry prior to medical school (the average score on this measure suggests students had “Some knowledge of biochemistry”). Lastly, students were fairly accurate in estimating what their score would be on the original exam if taken the day of the research exam (49 vs. 54%).

### Limitations

It could be argued that retention of biochemistry information, measured as performance on the same MCQs at two different time points is not the best way to assess retention, as students may simply recognize answers (and question stems) form the original exam, and that these cues jogged one’s memory for the correct answer without actually remembering the substance of the item. While we found no difference in performance on remembered vs. non-remembered items, implicit recognition of past items may have subtly biased the results. One way to reduce this bias would be to make minor changes to the question stem and/or answer choices so that the question is essentially the same in content, but less amenable to simple recognition by participants. One other potential limitation would be that because a free recall measure of performance was not employed during the initial summative exam we were unable to assess the change in free recall retention over time. Examination of free recall vs. cued recall retention may reveal the relative importance of one or the other for predicting subsequent high stakes exam performance. It would not be surprising to see that, because free recall likely taps into a better instantiated form of memory, free recall retention may end up being a better predictor of performance on the Step 1 exam.

### Future Directions

In this study we found that Biomedical knowledge taught during the first year of medical school is a good predictor of success on high-stakes exams, such as USMLE Step 1. However, this relationship should not be taken for granted across disciplines or organ systems. Future work should examine other course relationships in an effort to better understand the value of performing well throughout the pre-clerkship years as a means of predicting success on important standardized tests. Because free recall does not rely the assistance of seeing the answer embedded within a number of distractors, it represents a form of memory that relies both on availability of information in memory, but also accessibility of that information. The addition of a free recall measure of memory for some (or most) questions on summative exams would allow faculty to compare cued and free recall rates, and would allow faculty to assess the absolute free recall rates across modules. Free recall rates could be an indicator of how well modules are doing with making the information accessible, not just available to memory. It would also be interesting to see whether free recall rates predict student success in 3 ^rd^/4 ^th^ year clerkships, as students during their clerkships are required to have the knowledge they acquired during the pre-clerkship years available during rounds, and for making decisions regarding patient care.

## Conclusion

Enhancement of student retention of biomedical knowledge (“making it stick”) should be a primary goal for medical schools, either through adjustments to the curriculum or to pedagogical techniques. Although this study only provides one data point (biochemistry knowledge over a 10.5 month interval), the methods employed in this study are easily adapted to study cued and free recall across different modules, which would allow faculty to track year-to-year the changes in retention as a function of implemented improvements over time. Our findings also make a case for the incorporation of free recall measures of performance into summative exams. Because accurate free recall of information suggests a deeper, more instantiated, form of learning, and because it is a critical attribute of any medical professional, it may be beneficial to understand its predictive value for performance on board exams, but also for performance in non-didactic coursework, including 3 ^rd^ and 4 ^th^ year clerkships. We hope the findings of this study will serve as a guide to medical schools looking to improve their curricula using a fairly simple set of methods that may yield truly transformative results.

## Take Home Messages

• While it is not often assessed in the preclinical years, a student’s ability to access information through free recall, as opposed to cued recall through multiple choice questions, should be more closely studied. Opportunities for inclusion of free recall assessment in the pre-clinical years should be explored.
• Academically stronger students retain a greater percentage of originally learned material, and greater retention predicts performance on the USMLE Step 1 exam. While pedagogic strategies should be implemented to help all student improve their retention of pre-clinical knowledge, our data suggest that lower-performing students, especially, stand to benefit most from such strategies.

## Notes On Contributors

Anna Blenda*, PhD, is an Associate Professor of microbiology at University of South Carolina School of Medicine Greenville.

Renee Chosed*, PhD, is an Associate Professor of microbiology at University of South Carolina School of Medicine Greenville.

**Carrie Bailes**, MD, is a 2020 Graduate of the University of South Carolina School of Medicine Greenville.

**Mary Caldwell**, MD, is a 2020 Graduate of the University of South Carolina School of Medicine Greenville.

**Matthew Tucker**, PhD, is an Assistant Professor of neuroscience at the University of South Carolina School of Medicine Greenville.

* These authors contributed equally to this work.
