# Supplementary material for: Retention of Biomedical Knowledge in Medical Students Predicts USMLE Step 1 Scores
Source: MedEdPublish (2016). 2021 Mar 12;10:70. [Version 1] doi: 10.15694/mep.2021.000070.1 (PMC10939629; doi:10.15694/mep.2021.000070.1)
Supplement: Peer review history

# Reviewer report: Reviewer response for version 1

**DOI:** 10.21956/mep.18886.r26962

**Published:** 2021-06-16

**Authors:** Ken Masters

**Affiliations:** 1 Sultan Qaboos University

This review has been migrated. The reviewer awarded 3 stars out of 5 An interesting study indicating that biomedical knowledge in medical students predicts USMLE Step 1 scores.Overall, this is a useful study, but I would recommend that the authors make some changes in the presentation of the paper:• Much of the last paragraph in the Introduction should go into the Methods, as it describes the process.• I have to admit that I found great difficulty in reading and understanding the Methodology, and I think the problem is my unfamiliarity with the situation, but this would apply to the vast majority of readers. I think the main reason is that there is such a mixture of various examinations mentioned, frequently the same exam appears to be referred to by different names, that it took me 30 minutes to understand the process (and I’m still not entirely sure that I have it correct). For example, I THINK that the following terms all refer to the same exam:o “Foundations Summative exam”;o “original exam”; o “original summative exam”; o “original Biochemistry summative exam”;o “final exam of the Molecular & Cellular Foundations of Medicine (Biochemistry) module”, ando “original end-of-module exam”.If these are different, then the differences should be explained. If they are the same thing, then please choose one name, and use only that name all the way through the paper. (If the longer name is the official name, and the authors wish to use a shorter name, then please identify it first by the official, longer name, make it clear that it will be referred to by a shorter name, and then use only than shorter name). It might be a good idea to request someone who is unfamiliar with the process to re-read the Methodology. • Related to the Methodology, it is not clear when the students took the USMLE exam in relation to when they took the other exam/s. (As the USMLE an American exam, and the journal’s readership is international, familiarity with the US exam processes should not be assumed). The “prediction” would lead the readers to believe that the USMLE was taken after the other exam, but there appears to be a sense that it was before. So, clarity on this is required. (And, if before, then the use of the word “predict” should be addressed).So, an interesting study, but some issues need to be clarified. I look forward to Version 2 in which these issues are addressed.

Reviewer Expertise:

NA

No decision status is available

# Reviewer report: Reviewer response for version 1

**DOI:** 10.21956/mep.18886.r26963

**Published:** 2021-04-21

**Authors:** Barbara Jennings

**Affiliations:** 1 Norwich Medical School

This review has been migrated. The reviewer awarded 4 stars out of 5 In this well-designed study, the authors examine the retention of knowledge of biochemistry over a 10.5 month period as a proxy for how thoroughly the material was learned by medical student participants. In their introduction, the rationale for the project is made clear and the authors propose that retention rates can be used as a benchmark to develop the curriculum to support learners. They also explore the limitations of MCQ exams where information is cued to learners.The particular strengths of the article include (1) the clarity of the methods section and the logical study design described. (2) The comprehensive approach used to capture & analyse data about retained knowledge and participant confidence and motivation. The descriptive statistics used and the analyses of correlations were explained & presented clearly - but was the study adequately powered given the number of elements studied? Another aspect that could have been described more fully was recruitment, e.g. the study had IRB approval and I can see that there were 44 participants who were completing second year exams. But I am not clear how these were selected from the overall cohort or what the consent procedures were – I wondered about how recruitment to the study was affected by the statement in the methods “They were unaware of the nature and content of the test prior to participating?”The findings were interesting and in their conclusion the authors make some important suggestions about pedagogical and exam adjustments for medical curricula and the importance of intrinsic motivation was explored in the discussion.

Reviewer Expertise:

NA

No decision status is available
